# Chicago Medical Society

**Published:** 1881-08

**Authors:** 


### ARTICLE X.

CHICAGO MEDICAL SOCIETY. Stated Meeting June 20, 1881. Dr. E. Ingals, President in the Chair. Dr. L. H. Montgomery, Secretary.

A valuable paper on "Cremation," read by Dr. Chas. W. Purdy, will be found in this number of the MEDICAL JOURNAL AND EXAMINER.

The discussion was opened by Dr. G. C. Paoli, who said, that probably the greatest opposition to the adoption of cremation would come from the Orthodox Church, although he failed to see any antagonistic principle mentioned in the Scriptures. Such opposition had generally been made to any progress of science. Hence he called on the newspapers to enlighten the public on the subject, and impress the people favorably, for it was evident that grave-yards were very harmful, and that cremation had become a necessity.

Dr. R. E. Starkweather thought that cremation was soon to be adopted. A prominent bishop of the Church of England had lately expressed an opinion that it was only a matter of time. It was not, however, so urgent in this country as in the crowded cities of Europe. Monuments raised to the memory of the dead, he thought, were not so commendable as the foundation of charitable institutions and erection of buildings for that useful purpose.

Dr. C. T. Fenn thought the worship of the dead was so deeply rooted in the human heart that it was a question whether we could banish the idea that the dead is laid to sleep in the ground. He had no objection to have his body cremated, but he preferred to have those of his relatives and friends buried.

Dr. Valin said that the disposal of dead bodies was the mooted question. What shall we do with decomposing organic matter? He proposed to turn dead bodies into a source of revenue by selling them for dissection, and to soap manufacturers or fertilizing establishments.

Dr. E. Ingals did not approve of using the ashes of cremated bodies as fertilizers, but he knew of no better use to which old excavated bones could be put. A large quantity of such bones had come to the surface in the sand of old Lincoln Park grave-yard. He asked whether worms really infected dead bodies? He thought no living being could thrive in them. He referred to a pretty general fear among some people of being buried alive. Many people favored autopsies on that account. Many stories had been repeated of dead bodies having changed their position in the coffin; this was owing to the relaxation that followed rigor mortis, and should be explained to the laity. Besides a living being could not breathe in a common coffin, and could not live more than a few minutes. However, the body could not be considered dead before decomposition had commenced.

The decomposition by rotting or by cremating was the same, except that one process was much quicker. In order not to destroy criminal evidence some examination would probably be made. He did not believe there was any legal act preventing cremation. He disapproved of the extravagance generally displayed at funerals.

Dr. C. W. Purdy was convinced that the horror now manifested for incineration would be directed towards burying, as soon as the first had been witnessed a few times. He said worms did exist in corpses that decomposed with access to the air, but not in coffins underground. Yet serpents had been found in old coffins. He did not approve of the utilitarian theory. He professed a great respect for the dead, wichh he said should not be diminished, since it was one of the noblest feelings of the human heart, and he proposed cremation as a much more respectful procedure than burying.

The Society adjourned to October next. H. D. V.

---

### THE POISONOUS EFFECT OF INTRA-VENOUS INJECTIONS OF PANCREATIC MICROZYMES.

In a late communication to the French Academy of Sciences, A. Béchamp showed that all the known properties of the pancreas were dependent on its microzymes. Some experimenters, who had obtained toxic effects from the ferment of the pancreatic juice, desired to experiment with these microzymes, and see if their introduction into the organism would be followed by different results from those obtained in experimenting with pancreatine itself. They repeated their experiments with the microzymes of the liver, to have a point of comparison. They reached the following conclusions, which Dr. M. Raynaud read before the Academy of Medicine, March 8: 1. The injection into the blood, of isolated pancreatic microzymes, capable of digesting albuminoids, causes an almost instantaneous death, if their proportion is Og. 0001 for every kilogram in weight of the animal experimented upon (00 $\frac{1}{10,000,000}$ of its weight). It is impossible as yet to furnish a satisfactory explanation of the process of death, the only lesions observed being a congestion of the digestive mucous membrane, which ended in an effusion of blood, whenever death was relatively delayed. 2. The injection into the blood, of putrefied microzymes, which have evolved into bacteria, and are devoid of their fermentative power, does not give rise to any result. Hence the following important corollories: That death could not depend on embolism, which, beside, has never been found; that the bacteria replacing the pancreatic microzymes and the microzymes of fibrine, are absolutely harmless, and that a complete and radical change of function has been the result of the putrefaction experimentally induced in these micro-organisms. 3. The intra-venous injection with the microzymes of the liver is totally harmless, a fact which renders more wonderful the action of their congeners.

